# Relative Deprivation and Sickness Absence in Sweden

**DOI:** 10.3390/ijerph10093930

**Published:** 2013-08-29

**Authors:** Jonas Helgertz, Wolfgang Hess, Kirk Scott

**Affiliations:** 1 Department of Economic History and Centre for Economic Demography, Lund University, P.O. Box 7083, Lund 22007, Sweden; E-Mail: kirk.scott@ekh.lu.se; 2 Department of Economics and Centre for Economic Demography, Lund University, P.O. Box 7082, Lund 22007, Sweden; E-Mail: wolfgang.hess@nek.lu.se; 3 Demography Unit, Department of Sociology, Stockholm University, Stockholm 10691, Sweden

**Keywords:** sickness absence, Sweden, relative deprivation, duration analysis

## Abstract

*Background*: A high prevalence of sickness absence in many countries, at a substantial societal cost, underlines the importance to understand its determining mechanisms. This study focuses on the link between relative deprivation and the probability of sickness absence. *Methods*: 184,000 men and women in Sweden were followed between 1982 and 2001. The sample consists of working individuals between the ages of 19 and 65. The outcome is defined as experiencing more than 14 days of sickness absence during a year. Based on the complete Swedish population, an individual’s degree of relative deprivation is measured through income compared to individuals of the same age, sex, educational level and type. In accounting for the possibility that sickness absence and socioeconomic status are determined by common factors, discrete-time duration models were estimated, accounting for unobserved heterogeneity through random effects. *Results*: The results confirm that the failure to account for the dynamics of the individual’s career biases the influence from socioeconomic characteristics. Results consistently suggest a major influence from relative deprivation, with a consistently lower risk of sickness absence among the highly educated. *Conclusions*: Altering individual’s health behavior through education appears more efficient in reducing the reliance on sickness absence, rather than redistributive policies.

## 1. Introduction

A successful strain of research within public health and health economics over recent decades has concerned the extent to which differences in socioeconomic status lead to different health outcomes. The general consensus today is that the socioeconomic gradient plays an important role in health outcomes and that this role appears robust over time and across countries [1,2]. The effects of uneven income distribution and relative income of the individual are also assumed to be of importance for health, as suggested by several studies (see [3] for a review and, also, [4]).

This study will continue this line of research through focusing on relative deprivation as a driver of poor health, but with a slightly more refined approach. Most studies using relative income attempt to identify appropriate reference groups against which individuals measure themselves. This construction of reference categories is difficult and often lacking clear motivation. Using register data for Sweden, we construct relative income with unprecedented precision, measured as the relationship between an individual’s income and the mean income for all individuals in Sweden characterized by the same age, sex and education (defined by individual study programs and lengths). We then investigate the effect of relative income on sickness absence from work.

### The Swedish Context

Recent years have given rise to heated public debate within the Swedish political and journalistic realms regarding the pressure that increasing sickness absence has placed upon the health of the welfare state. The societal costs of sickness absence have been growing, and in 2008, roughly 10% of the Swedish population in working ages were absent from work during a consecutive period of at least fifteen days. While this share may not appear alarming at first glance, the numbers behind this modest figure are staggering.

The Swedish transfer system currently allows for individuals to receive compensation at a rate of 80% of their income from the third day of absence. During the first 14 days, compensation is paid by the employer, with the Swedish Social Insurance Agency taking responsibility for payments from day 15. This two-payer construction means that we have very little information on sickness absence periods lasting less than 15 days, since they are not included in the official statistics. When restricting focus to those absent from work for at least 15 consecutive days, the Swedish government paid out compensation for approximately 45 million sick days in 2008 [5]. Thus, we can begin to see the enormity of the issue, amounting to roughly 195,000 working years being lost due to extended sickness absence. This figure is calculated based on a working year consisting of 230 days, calculated at 47 five-day weeks minus holidays. The actual number of workdays falls between 226 and 229, depending on when holidays occur.

Since compensation for sickness absence from the tax-financed Swedish Social Insurance Agency is only paid out from day 15 and the Social Insurance Agency registered 550,000 new cases of individuals receiving sickness benefits in 2008, we need to add an additional 33,000 person-years to account for the 14 days of employer-financed sick leave during the initial qualification period. (Calculated as (544, 708 × 14)/230). This brings us to a grand total of 228,000 person-years lost to sickness absence during a single calendar year. Furthermore, this must be seen as a lower bound, as all shorter periods of sickness absence (less than 15 consecutive days) are excluded from this figure. Naturally, this not only represents a massive burden on society in terms of a sizeable share of the population suffering from poor health, but it also implies a loss in terms of forgone labor, as well as a large tax burden related to sickness benefit payments.

Recently, a number of changes in the rules regarding eligibility for sickness benefits have been implemented. These changes have had the desired effect of decreasing the number of people receiving benefits, but have also been heavily criticized in the press for being too heavy-handed. Arguably, the most valid criticism of these policy changes is that they have been implemented with no real understanding of why people are absent from work and, therefore, do not ameliorate the problems underlying the high levels of sickness absence. This article will therefore examine some of the key factors driving sickness absence.

The objective of this paper is to analyze the effects of relative income, absolute income and educational attainment on the propensity of becoming absent due to sickness from work. Towards this aim, discrete-time duration models are used, which facilitate the estimation of income and education effects, while at the same time controlling for various socioeconomic factors, as well as the duration of uninterrupted employment until transition to sickness absence (employment duration). Controlling for employment duration is important, as it is strongly related to both income and the propensity of becoming absent due to sickness. Failing to account for duration will therefore produce bias in the estimated income effects.

## 2. Background

Primarily being considered as an indicator of morbidity, sickness absence has been observed to be positively associated with several other health outcomes, including mortality risk. Vahtera *et al.* [6] find a positive association between longer sickness absence periods and subsequent mortality from cardiovascular disease and cancer. Such findings would corroborate that sickness absence is indeed an indicator of poor health. Furthermore, as indicated by several studies, sickness absence is likely to be an underestimation rather than an overestimation of the actual prevalence of poor health in the workplace (see, for example, [7,8,9]). Consequently, a better understanding regarding the generation of health inequalities among working individuals appears to be of great importance, for the individual, as well as for the society as a whole.

Similar to a range of other health outcomes, a strong socioeconomic gradient in terms of the propensity to be absent due to sickness has been observed in previous research. The explanations as to why socioeconomically disadvantaged individuals suffer from higher sickness absence propensities range from lower access to healthcare and goods to maintaining less healthy diets and less exercise habits [10,11]. The Swedish context—similar to numerous other welfare states—is, however, characterized by universal health care, as well as a broad range of subsidized pharmaceuticals. As a consequence, the link between socioeconomic status and health appears unlikely to be strongly related to differential access to healthcare. Therefore, behavioral differences across socioeconomic strata, frequently unobserved in empirical data, appear more likely to be the underlying cause of the persistent socioeconomic gradient in sickness absence. However, studies that control for various behavioral characteristics, such as smoking and working conditions, nevertheless fail to reject the socioeconomic health gradient [12]. This suggests that benefits enjoyed as a consequence of a more elevated socioeconomic position are positively affecting the individual’s health, thereby leading to a negative influence on the propensity to be absent due to sickness.

While sickness absence is primarily viewed as a consequence of actual poor health, and, thereby, an indicator of morbidity, empirical evidence from numerous contexts suggests a substantial part of unscheduled absence from work as voluntary, or shirking [13,14,15]. This is also likely to be one of the factors that underlie the socioeconomic gradient in sickness absence. More specifically, individuals in the lower end of the socioeconomic distribution are more likely to find themselves in work positions that are unrewarding in terms of job tasks, career opportunities and financial remuneration. A particularly relevant dimension to consider as regards all aforementioned aspects refers to the individual’s own expectations regarding a future labor market career. More specifically, an individual completing medical school is likely to have very different expectations regarding future income and working conditions than someone ending the educational career after the mandatory nine years of primary schooling. According to the relative deprivation hypothesis, the individual’s relative performance should therefore be the focal point of interest (see, for example, [16,17]). In contexts where a minimum standard of living is guaranteed—regardless of earned income—the individual’s relative position is furthermore assumed to become increasingly important. Individuals experiencing worse labor market returns than a relevant comparison group are—according to the hypothesis—increasingly likely to be discontent with their work situation, thereby increasing the propensity of unscheduled absence from work.

In the literature, the relative deprivation approach frequently measures the general income inequality in an area, region or country, often operationalized through the Gini coefficient or some other measure at a fairly aggregate level. While many arguments have been made for the importance of the wider population for an individual’s sense of well-being [18,19,20,21], another important dimension is found in more individualistic measures of relative deprivation, which may capture the aspirations of individuals and, thereby, the degree to which they may feel they have underperformed.

Of paramount importance to the conclusions of this article is the ability to separate the influence of the individual’s degree of relative deprivation from that resulting from other measurements of socioeconomic status (SES), including the individual’s absolute income level, as well as educational attainment. While the link between SES and health has been identified in numerous studies, there appears to exist a considerable degree of overlap in terms of the influence of each respective component of SES. More explicitly, attained education is believed to influence health, both directly and indirectly. Theoretically, the direct and independent influence should manifest itself largely through lifestyle differences. Highly educated individuals are characterized by a greater ability to identify and process information related to health-promoting, as well as hazardous behavior. This has been supported by empirical research showing the more highly educated being less prone to obesity and heavy smoking and drinking, as well as more likely to exercise regularly [22].

The indirect influence of education on health may be partly found in characteristics of the individual’s occupation. Occupations demanding advanced formal skills are considered to be associated with greater rewards, ranging from a greater sense of control and independence to greater employment security during economic downturns. Consequently, higher educational attainment should be associated with better health, due to a higher occupational status, regardless of the degree of relative deprivation [23,24]. If this is true, more highly-educated individuals should benefit from better health, even after controlling for absolute and relative income. As relative deprivation increases, it remains unclear on theoretical grounds whether the health advantage of the more highly educated changes. Potentially, based on the differing life expectations characterizing individuals of different educational levels, the extent of psychosocial stress associated with a failure to perform could increase disproportionately among those with higher education.

While correlated with individual educational attainment, but operating indirectly rather than directly, differences in absolute income attainment are potentially also linked to poor health. While income is not a primary factor determining access to healthcare in Sweden, it may still have an effect on health, since resources are important in determining the individual’s exposure to various environmental factors, such as poorer quality housing, disadvantaged neighborhoods and possible residential crowding. On the other hand, there also exists support for the idea that increasing economic resources may not lead to greater health and may actually lead to worse health. The mechanism here is related to an increased ability to invest in hazardous health behavior [25,26,27].

Of particular interest to this article is the influence of relative, rather than absolute, income, since this is believed to be an independently important health-generating mechanism. Theory postulates that an unfavorable position in the relative income distribution is linked to exposure to psychosocial stress, which negatively impacts health [19,28,29,30]. Hence, being relatively deprived, measured as having an income that is substantially lower than one’s peers, should be indicative of experiencing a considerable degree of stress, due to relative underperformance.

## 3. Data, Variables and Econometric Method

### 3.1. Data and Sample

The sample examined in the article is selected from the Swedish Longitudinal Immigrant (SLI) database, administered at the Centre for Economic Demography, Lund University, Sweden. The database contains longitudinal individual level information on roughly 550,000 native born Swedes and foreign born individuals between 1968 and 2001. Information is obtained from various administrative registers, gauging a range of individual level characteristics; demographic, socioeconomic and health-related. The study population is restricted to working individuals in the ages between 19 and 65. The transition of interest is that from work to sickness absence, implying that the individual is only considered to be at risk of sickness absence during years when labor-related earnings exceed three base amounts. (The “base amount” is a purely administrative measure, but since most public transfers in Sweden are related to that amount, we choose to use it as the basis for our determination of being established in the workforce. Three base amounts in 2013 amounted to SEK 133,500 or roughly USD 20,000). The sample selected for the analysis consists of approximately 184,000 individuals, followed on a yearly basis during the time period, 1982–2001. The sample includes both males and females, consisting of native Swedes (52%) and of immigrants from the 16 largest immigrant nationalities in Sweden.

### 3.2. Dependent Variable

Using data from the income register, the amount of sickness absence benefits received during a calendar year is used to define the dependent variable for the analysis. Of explicit interest is whether a working individual experiences a period of sickness absence exceeding 14 days during any given calendar year. The motivation for the threshold pertains to changes over time in the system governing compensation from sickness absence. Prior to 1992, eligibility for sickness benefits was conditional on first completing a qualifying period of between zero to two days, during which the individual received no compensation. Subsequently to this, the individual received sickness benefits from the government until either returning to work or *de facto* permanently exiting the labor force through disability pension retirement. Due to the amount of sickness absence benefits received being directly linked to the individual’s earnings, the number of sick days experienced during one year can be calculated with considerable precision. After 1992, this procedure becomes more complicated, as the receipt of sickness absence benefits can only be used to determine that the individual experienced a period of sickness absence of at least 15 consecutive days during these years. (The changes in the system governing sickness absence also made the employer responsible for providing the employee with sickness benefits up until day 15, something which is not observable in the data.) In order to use a consistent definition of sickness absence throughout the entire period that is examined, a working individual experiencing at least 15 days of sickness absence during any given year is considered to make the transition from work to sickness absence. In the analysis, an individual who during two or more consecutive years experiences the event is considered to experience an equal number of incidences of sickness absence. Using this definition, 427,000 sickness absence events are recorded in the data. In order to investigate to what extent the results depend on this definition, in the sensitivity analysis, consecutive years of experiencing sickness absence exceeding the threshold of 14 days were considered as one single event.

### 3.3. Independent Variables

#### 3.3.1. Relative Income

The key independent variable used in the analysis gauges an individual’s labor market performance in terms of their relative income. Using data on the entire Swedish working age population, the mean income was calculated for all combinations of sex, age, educational level (primary/secondary/university) and educational type (e.g., law/economics/business administration). Consequently, the benchmark income indicates the expected labor market returns for an individual with such characteristics. The individual’s pre-tax income from work is henceforth used to calculate the individual’s income as a share of the relevant benchmark income. With a value of one indicating an income that is identical to the benchmark, over 90% of the observations in the sample are recorded, with a relative income ranging between 0.6 and 1.4. The data reveal a high concentration of individuals around the mean (0.96), hence enjoying an income similar to what could be expected, given the individual’s age, sex and education. Due to the absence of well-grounded *a priori* expectations regarding the (functional form of the) effect of relative income on the sickness absence hazard, the variable is expressed as vigintiles (groups each containing 5% of the sample distribution) in the analysis. This ensures a flexible specification of the relative income effect.

#### 3.3.2. Other Variables

Also included in the models are individual level control variables, such as age, country of origin, time since migration and civil status. Additionally, the models control for the regional unemployment rate, in previous research indicated as a predictor of sickness absence. Aiming to identify the influence from relative income on the probability of sickness absence, the previous section outlined the necessity to separate absolute from relative income. Therefore, the models also control for the individual’s absolute income, expressed as integer base amounts and coded as a set of dummy variables. As the base amount is adjusted on a yearly basis to account for inflation, the measurement used should be interpreted as the individual’s absolute real income. The two measures of income display a positive relationship, but only with a correlation coefficient around 0.6. The influence of absolute income—similar to relative income—is, in more extended models, interacted with the individual’s educational attainment. This allows for behavioral differences between individuals of different educational levels in their response to absolute or relative income. Moreover, in investigating the dynamics of sickness absence patterns, an additional set of variables were constructed from the data. Firstly, a set of dummy variables control for the uninterrupted duration of the current employment spell. Secondly, another variable gauges the duration of the preceding employment spell. Lastly, by controlling for the sequence in which the actual spell occurs, the models distinguish between individuals with comparatively stable and those with more volatile employment patterns.

### 3.4. Econometric Method

As mentioned briefly in the Introduction and discussed in detail in Section 4.1 below, both (relative) income and the propensity of becoming absent due to sickness are strongly affected by the duration of uninterrupted employment. Therefore, to avoid bias in the estimated income effects, we resort to duration models when analyzing the impact of income on the sickness absence hazard. Since the duration times until transiting into sickness absence are substantially grouped into yearly intervals, discrete-time hazard models are employed to analyze the data at hand. These models can be estimated using conventional regression models for binary response panel data, which are implemented in all common statistical software packages. This approach is very appealing, due to its computational simplicity and due to the fact that estimation results can be compared straightforwardly with those obtained in previous studies on individual sickness absence probabilities (see, for example, [31]). Moreover, unlike continuous-time methods, discrete-time duration models do not require special extensions to deal with tied failure times, and they can easily be extended to account for unobserved heterogeneity, even if the number of observations is large.

Having motivated the use of discrete-time hazard models in the empirical analysis, these methods are now introduced in more detail. Let *T_j_* be a non-negative random variable measuring the time until sickness absence for separate spells, *j* =1,..., *J*, of uninterrupted employment. In a discrete-time framework, the core of duration analysis is formed by the probability that sickness absence occurs in a given time interval, [*t_k_*,*t_k_*_+1_), *k* =1, 2, . . .,*k^max^* and *t*_1_ = 0, conditional on not having occurred up to the beginning of the interval and given the explanatory variables included in the regression model. This conditional probability is termed the discrete-time hazard rate and is formally defined as:

*h_jk_* := *P*(*T_j_* < t*_k+1_*|T*_j_* ≥ *t_k_*,***x****_jk_*) = *F*(*γ_k_* + ***x***′*_jk_β*) (1)

where *γ_k_* is a function of (interval) time that allows the hazard rate to vary across durations (somewhat loosely, *γ_k_* will be referred to as the grouped-duration baseline hazard, although this is not formally correct in all instances; only if *γ_k_* is modeled as an interval-specific step function and if *F* (·) is the extreme value minimum distribution, *γ_k_* can be shown to be the exact grouped-duration analogue of the continuous-time Cox [32] baseline hazard (see for example [33,34] for a formal proof)), ***x****_jk_* is a vector of possibly time-varying covariates and *F* (·) is an appropriate response function ensuring that 0 ≤ *h_jk_* ≤ 1 for all *j*, *k*.

For each employment spell, the last observed year in which the spell is in progress can be recorded. In the following, this terminal year is denoted *K_j_*, the subscript, *j*, indicating that it may differ across spells. The spell is complete if a sickness absence period is observed during the *k*^th^ year and right-censored, otherwise. Introducing a binary variable, *y_jk_*, taking the value one if a spell is observed to cease during the *k*^th^ time interval and zero, otherwise, the spell’s contribution to the likelihood function is given by:

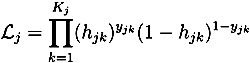



Assuming conditional independence between spells, the likelihood for the whole sample is:

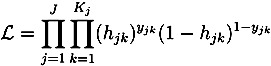

with the corresponding log-likelihood function:

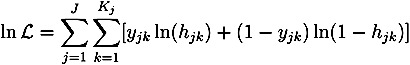
(2)


This expression is structurally isomorphic to a standard log-likelihood function for a binary regression model with dependent variable, *y_jk_*. (To obtain consistent parameter estimates from this log-likelihood, spells must be independent, censoring must occur only at interval boundaries and censoring must not provide any information about *T_j_* beyond that available in the covariates (see, for example, [35,36,37] for excellent surveys on the derivation of the likelihood).) To be able to estimate the model parameters, a functional form for the hazard rate, *h_jk_*, needs to be specified. The most commonly encountered functional specifications are the normal, logistic and extreme-value minimum distribution, leading to a probit, logit or cloglog model, respectively.

The specification of the hazard rate in Equation (1) implicitly assumes that all factors affecting the hazard rate can be observed. In practice, however, it will rarely be the case that all sources of variation in the hazard rate are exhausted by the observed explanatory variables included in the model. Instead, unobserved sources of heterogeneity are very likely to exist in addition to the differences that are explicitly modeled via the inclusion of measured covariates. It has long been known that neglecting these unobserved factors can lead to a serious bias in the estimated hazard rates (see, for example, [38,39,40] for early discussions of this issue). To avoid bias in the estimated hazard rates, unobserved heterogeneity can be accounted for by including Gaussian random effects into the binary choice model framework above. Nicoletti and Rondinelli [41] have shown that using normal random effects works well, even if the true distribution of the random effects is not Gaussian.

## 4. Empirical Analysis: The Duration until Sickness Absence

### 4.1. Descriptive Statistics

Table 1 shows summary statistics for the entire sample and grouped by educational level. The table reveals higher average absolute incomes enjoyed by individuals with higher educational attainment, here expressed as integer base amounts. Furthermore, individuals with lower educational attainment appear to experience a higher incidence of sickness absence, indicated by a higher number of spells, as well as a shorter duration of employment between each period of sickness absence. This could be indicative of a number of factors, including worse health habits among individuals with less schooling, as well as poorer working conditions in occupations that typically should be characterized by a lower degree of independence and control, as well as a greater prevalence of physically strenuous tasks.

The overall incidence of sickness absence is consistent with the development over time in Sweden as a whole. More specifically, the period examined in this article can be divided into two distinctly different periods, where the 1980s were characterized by considerably higher and generally increasing rates of sickness absence than the 1990s. The lower incidence of sickness absence during the 1990s was caused by the Swedish economic crisis during the early years of the decade, causing substantial changes in the system governing sickness absence compensation.

**Figure 1:**
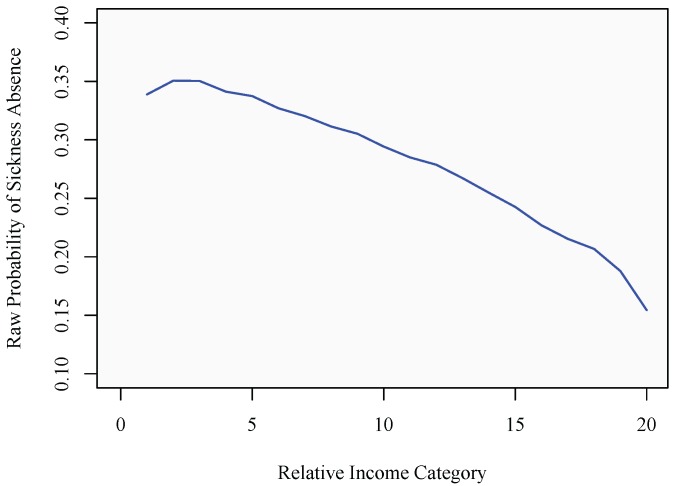
Relative income and sickness absence.

One empirical contribution of this article pertains to the use of duration analysis in investigating the mechanisms underlying the relationship existing between an individual’s labor market performance and the propensity for sickness absence. Figure 1 tentatively indicates the existence of such a relationship, where relatively deprived individuals, on average, display a considerably higher raw probability of sickness absence. Evidently, there are several potential explanations to an individual’s position in the relative income distribution. To the extent that one’s position in the relative income distribution is synonymous with increased work-related stress and general discontent with one’s situation, this could possibly have adverse health consequences, thereby explaining the observed pattern. However, the greater propensity of sickness absence among the lowest performing individuals could also be linked to a greater incidence of shirking.

**Table 1:** Descriptive statistics: full sample and grouped by educational level.

	Full sample			Primary education			Secondary education			University education		
	Mean	Min	Max	Mean	Min	Max	Mean	Min	Max	Mean	Min	Max
Relative income	0.964	0.001	4.597	0.975	0.047	4.597	0.972	0.006	4.591	0.939	0.001	4.597
Absolute income	4.911	3	76	4.450	3	28	4.643	3	49	5.803	3	76
*Education:*												
Primary	0.250	0	1	1.000	0	1	0.000	0	1	0.000	0	1
Secondary	0.477	0	1	0.000	0	1	1.000	0	1	0.000	0	1
University	0.273	0	1	0.000	0	1	0.000	0	1	1.000	0	1
Gender (female = 1)	0.487	0	1	0.439	0	1	0.477	0	1	0.549	0	1
Civil status (1 = married)	0.480	0	1	0.515	0	1	0.439	0	1	0.520	0	1
*Age*	38.07	18	65	40.10	18	65	36.64	18	65	38.70	18	65
Age squared	1,564	324	4,225	1,733	324	4,225	1,457	324	4,225	1,595	324	4,225
Years since migration	6.121	0	65	7.372	0	65	5.780	0	61	5.569	0	63
Years since migration squared	121.1	0	4,225	144.3	0	4,225	116.3	0	3,721	108.1	0	3,969
Metropol.residence	0.485	0	1	0.465	0	1	0.457	0	1	0.553	0	1
Local unemployment rate	6.100	0.160	24.02	5.748	0.160	24.02	6.212	0.160	24.02	6.227	0.160	24.02
Year	1993	1982	2001	1992	1982	2001	1993	1982	2001	1994	1982	2001
Duration	2.494	1	20	2.063	1	20	2.464	1	20	3.168	1	20
Lag duration	1.369	0	19	1.279	0	19	1.384	0	19	1.459	0	19
Number of spells	2.953	1	6	3.183	1	6	3.043	1	6	2.543	1	6
*Spell duration conditional on the spells ending in sickness absence:*												
1st spell	2.551	1	20	2.165	1	20	2.543	1	20	3.200	1	20
2nd spell	1.986	1	19	1.747	1	19	1.993	1	19	2.375	1	18
3rd spell	1.756	1	18	1.589	1	17	1.774	1	18	2.016	1	18
Observations	1,525,310			381,839			727,438			416,033		
Spells	611,599			185,094			295,180			131,325		
Individuals	184,494			50,854			85,952			47,688		

Note: Average durations are calculated per spell, and the average number of spells is calculated per individual. All other average values are calculated per observation.

**Figure 2:**
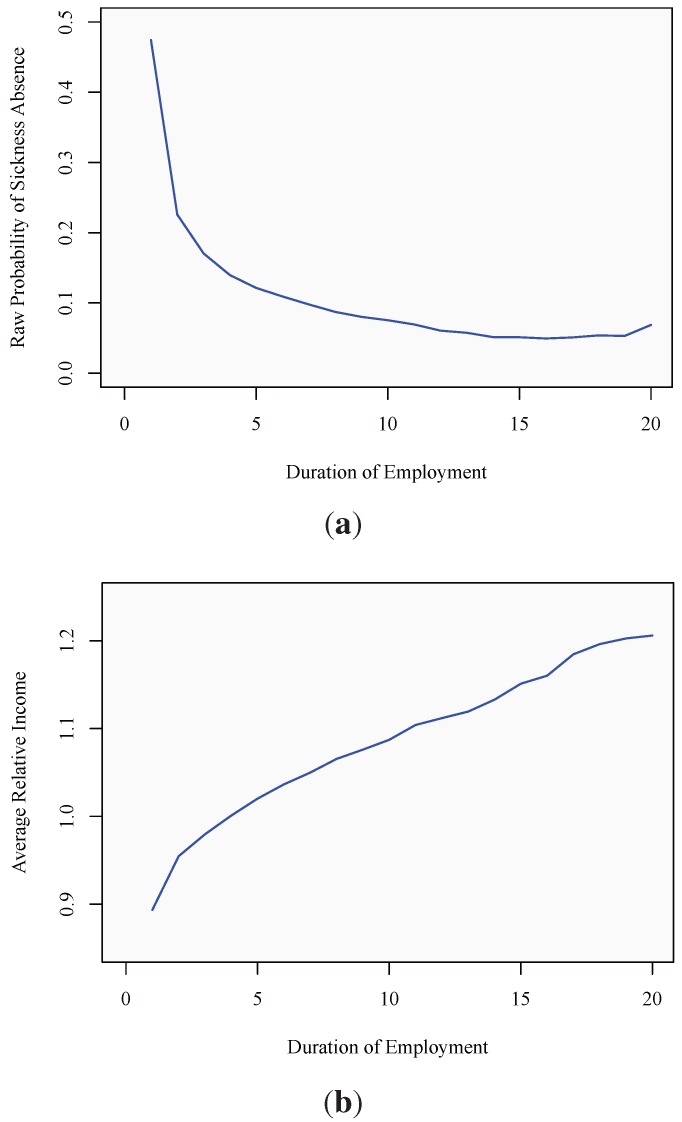
The effect of duration. (**a**) Duration and sickness absence; (**b**) duration and relative income.

In order to properly identify the influence from relative income on the sickness absence propensity, it is important to recognize the mechanisms generating the individual’s position in the relative income hierarchy. More specifically, it is possible that sickness absence propensity and relative income attainment are jointly determined by some third factor. In illustrating this possibility, panel (a) in Figure 2 displays the unconditional relationship between the duration of an employment spell and the raw propensity to become absent due to sickness. As is evident, the propensity of sickness absence decreases with the duration of the employment spell, suggesting that incidences of sickness absence are recurrent and, thereby, negatively affecting the duration of employment spells among individuals with a higher propensity to be sick. Turning to panel (b), additional evidence is provided for the importance of taking the duration of employment spells into account. Individuals enjoying longer uninterrupted spells of employment on average also perform better in terms of relative income attainment, likely to be due to being positioned on a more favorable career trajectory. As a consequence, the negative relationship between income and sickness absence could therefore, at least partly, result from individuals who are disproportionately allocated to shorter durations of employment being characterized by certain characteristics.

### 4.2. Regression Analysis

In order to account for the simultaneous correlation of an ongoing work spell’s duration with the probability of being absent due to sickness and the attained relative income, the multivariate analysis relies on discrete-time duration models. The purpose of this modeling strategy is to obtain unbiased estimates of the relationship between relative income and sickness absence. As discussed in Section 3.4, discrete-time duration models can be estimated using the standard binary choice model framework for panel data. This makes it possible to directly compare the results obtained from duration models with those obtained from conventional binary choice models used in previous studies on transitions into sickness absence. In fact, the previously used binary choice models can be regarded as discrete-time duration models, where the baseline hazard is restricted to be constant, implying that the sickness absence probability is forced to be independent of the duration of the current employment spell.

Table 2 displays regression coefficients from discrete-time duration models employing a logit link function and Gaussian random effects to account for unobserved individual heterogeneity. Although accounting for unobserved individual heterogeneity is common practice in micro-panel studies, it is of particular importance in the context of this study. It has long been known in the literature that neglecting unobserved heterogeneity in duration models causes spurious negative duration dependence in the estimated hazard function (see, for example, [40]). Since, as shown in Figure 2 above, both the relative income and the hazard of becoming absent due to sickness depend on the duration of uninterrupted employment, a failure to correctly estimate the effect of duration on the hazard will also cause bias in the estimated effect of relative income. In order to correctly evaluate the effect of relative income on the sickness absence propensity, it is, therefore, essential to control for unobserved individual characteristics.

Model 1 is similarly specified as the models encountered in the previous literature, without any controls for characteristics pertaining to the individual’s employment and sickness absence history. Apart from the variables of paramount interest for this article, the relative income vigintiles, the model includes a comprehensive set of individual and contextual covariates, including the individual’s position in the absolute income distribution. The results are largely consistent with previous findings and *a priori* expectations, suggesting substantially lower probabilities of transiting into sickness absence among the highly educated and among men. Contrary to expectations, the results suggest a link between absolute income and the sickness absence hazard, where increased income attainment is related to elevated odds of becoming absent due to sickness. This result only emerges when simultaneously controlling for the individual’s position in the relative income distribution, thus indeed suggesting different mechanisms linking these two aspects of an individual’s SES and the risk of sickness absence. The effect of relative income, however, is virtually unaffected by the inclusion or exclusion of absolute income. Recalling the essentially nonexistent link between financial resources and access to healthcare in Sweden, this result indicates that increasing economic resources—if anything—cause an increase in the consumption of goods that are detrimental to the individual’s health. In order to better understand these mechanisms, extended models will interact absolute income attainment and the individual’s educational level.

**Table 2:** Estimations of the discrete-time hazard of being absent due to sickness in a given year: logit models with Gaussian random effects.

	Baseline analysis				Robustness analysis	
	Model 1	Model 2	Model 3	Model 4	Model 5	Model 6
Secondary education	−0.4787	−0.4146	−0.3852	−0.3357	−0.2964	−0.4690
	(0.000)	(0.000)	(0.000)	(0.000)	(0.000)	(0.000)
University education	−1.5668	−1.3951	−1.2115	−0.9829	−0.8198	−1.2607
	(0.000)	(0.000)	(0.000)	(0.000)	(0.000)	(0.000)
Gender	1.0991	0.9924	0.9164	0.9380	0.7972	0.8653
	(0.000)	(0.000)	(0.000)	(0.000)	(0.000)	(0.000)
Metropolitan dummy	0.1042	0.0958	0.0657	0.0650	0.0789	0.1177
	(0.000)	(0.000)	(0.000)	(0.000)	(0.000)	(0.000)
Unemployment rate	0.0088	0.0088	0.0095	0.0095	0.0076	0.0078
	(0.000)	(0.000)	(0.000)	(0.000)	(0.000)	(0.006)}
Age	−0.1235	−0.0888	−0.1259	−0.1299	−0.0928	−0.0899
	(0.000)	(0.000)	(0.000)	(0.000)	(0.000)	(0.000)
Age squared	0.0019	0.0015	0.0017	0.0018	0.0012	0.0012
	(0.000)	(0.000)	(0.000)	(0.000)	(0.000)	(0.000)
Years since migration	0.0108	0.0184	−0.0144	−0.0149	−0.0200	0.0042
	(0.000)	(0.000)	(0.000)	(0.000)	(0.000)	(0.113)
Years since migration squared	−0.0003	−0.0004	0.0001	0.0001	0.0003	−0.0002
	(0.000)	(0.000)	(0.001)	(0.001)	(0.000)	(0.008)
Civil status	0.0263	0.0199	0.0044	0.0064	0.0098	0.0738
	(0.000)	(0.003)	(0.479)	(0.299)	(0.134)	(0.000)
*Relative income categories (13 = reference category)*						
Category 1	1.2968	1.1267	1.1422	1.4450	1.4082	1.1718
	(0.000)	(0.000)	(0.000)	(0.000)	(0.000)	(0.000)
Category 2	0.9920	0.8611	0.8673	1.1585	1.0513	0.8999
	(0.000)	(0.000)	(0.000)	(0.000)	(0.000)	(0.000)
Category 3	0.8535	0.7486	0.7498	0.9686	0.8633	0.7360
	(0.000)	(0.000)	(0.000)	(0.000)	(0.000)	(0.000)
Category 4	0.7109	0.6226	0.6224	0.7760	0.6723	0.5486
	(0.000)	(0.000)	(0.000)	(0.000)	(0.000)	(0.000)
Category 5	0.6218	0.5506	0.5476	0.6791	0.5613	0.4436
	(0.000)	(0.000)	(0.000)	(0.000)	(0.000)	(0.000)
Category 6	0.5280	0.4721	0.4679	0.5991	0.4882	0.4504
	(0.000)	(0.000)	(0.000)	(0.000)	(0.000)	(0.000)
Category 7	0.4614	0.4143	0.4080	0.5505	0.4736	0.4484
	(0.000)	(0.000)	(0.000)	(0.000)	(0.000)	(0.000)
Category 8	0.3873	0.3521	0.3473	0.4523	0.3729	0.4181
	(0.000)	(0.000)	(0.000)	(0.000)	(0.000)	(0.000)
Category 9	0.3289	0.2997	0.2961	0.3957	0.3202	0.4052
	(0.000)	(0.000)	(0.000)	(0.000)	(0.000)	(0.000)
Category 10	0.2353	0.2171	0.2131	0.2634	0.2128	0.2106
	(0.000)	(0.000)	(0.000)	(0.000)	(0.000)	(0.000)
Category 11	0.1498	0.1371	0.1345	0.2227	0.1716	0.2237
	(0.000)	(0.000)	(0.000)	(0.000)	(0.000)	(0.000)
Category 12	0.0864	0.0820	0.0814	0.1122	0.0868	0.0749
	(0.000)	(0.000)	(0.000)	(0.000)	(0.004)	(0.142)
Category 14	−0.1020	−0.0949	−0.0898	−0.0872	−0.0837	−0.0961
	(0.000)	(0.000)	(0.000)	(0.001)	(0.006)	(0.066)
Category 15	−0.1792	−0.1639	−0.1584	−0.1487	−0.1455	−0.2091
	(0.000)	(0.000)	(0.000)	(0.000)	(0.000)	(0.000)
Category 16	−0.2785	−0.2560	−0.2472	−0.2653	−0.2264	−0.2207
	(0.000)	(0.000)	(0.000)	(0.000)	(0.000)	(0.000)
Category 17	−0.3620	−0.3302	−0.3183	−0.3536	−0.3141	−0.3479
	(0.000)	(0.000)	(0.000)	(0.000)	(0.000)	(0.000)
Category 18	−0.4196	−0.3830	−0.3701	−0.4468	−0.3829	−0.3854
	(0.000)	(0.000)	(0.000)	(0.000)	(0.000)	(0.000)
Category 19	−0.5529	−0.5018	−0.4877	−0.6670	−0.5504	−0.5450
	(0.000)	(0.000)	(0.000)	(0.000)	(0.000)	(0.000)
Category 20	−0.6201	−0.5687	−0.5530	−0.8326	−0.7442	−0.8147
	(0.000)	(0.000)	(0.000)	(0.000)	(0.000)	(0.000)
*Absolute income in base amounts (1 and 2 are excluded from the sample; 4 = reference category)*						
3	−0.3631	−0.4259	−0.3846	−0.4907	−0.1780	−0.4296
	(0.000)	(0.000)	(0.000)	(0.000)	(0.000)	(0.000)
5	0.1604	0.1951	0.1773	0.2614	0.1339	0.1750
	(0.000)	(0.000)	(0.000)	(0.000)	(0.000)	(0.000)
6	0.2579	0.3134	0.2916	0.4569	0.2632	0.2423
	(0.000)	(0.000)	(0.000)	(0.000)	(0.000)	(0.000)
7	0.3425	0.4035	0.3846	0.5946	0.3487	0.2715
	(0.000)	(0.000)	(0.000)	(0.000)	(0.000)	(0.000)
8–9	0.3655	0.4260	0.4194	0.6975	0.4543	0.3922
	(0.000)	(0.000)	(0.000)	(0.000)	(0.000)	(0.000)
≥10	0.2122	0.2673	0.3017	0.4653	0.1717	−0.3442
	(0.000)	(0.000)	(0.000)	(0.000)	(0.043)	(0.043)
Country dummies	yes	yes	yes	yes	yes	yes
Year dummies	yes	yes	yes	yes	yes	yes
Duration dummies	no	yes	yes	yes	yes	yes
Spell dummies	no	no	yes	yes	yes	no
Lagged duration	no	no	yes	yes	yes	no
Income-education interactions	no	no	no	yes	yes	yes
*ρ*	0.4240	0.3377	0.2425	0.2420	0.1622	0.3360
	(0.000)	(0.000)	(0.000)	(0.000)	(0.000)	(0.000)
Observations	1,525,310	1,525,310	1,525,310	1,525,310	1,322,498	592,405
Spells	611,599	611,599	611,599	611,599	408,787	184,494
Individuals	184,494	184,494	184,494	184,494	184,494	184,494
Log likelihood	−719,085	−708,801	−702,840	−702,317	−545,275	−240,238

*Note*: *p*-values are given in parentheses. All models include individual-specific random intercepts, where *ρ* denotes the fraction of the error variance that is due to variation in the unobserved individual factors. More precisely, 
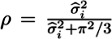
, where 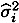 the estimated variance of the individual-specific random intercepts and *π*^2^/3 is the variance of the logistic distribution. In Models 1–3, the reported coefficients on the relative and absolute income categories are averages over the complete sample, whereas the respective coefficients in Models 4–6 are averages over the baseline category of individuals with primary education only.

Turning to the relative income categories, a quite expected relationship between the individual’s degree of relative deprivation and their probability of sickness absence emerges. Compared to an otherwise similar individual with an income equal to the average for a given age, sex and education (denoted in the table as relative income category 13), the odds of sickness absence among the lowest three vigintiles are, in a steadily increasing fashion, between 135 and 266% higher (corresponding to *β*-coefficients of about 0.85 and 1.30, respectively). For the highest vigintile, the odds of becoming absent due to sickness are, rather, 46% lower (*β* ≈−0.62) than for those facing no relative deprivation. Furthermore, the narrow confidence intervals (95%, not shown) around the estimates generally suggest that the relative income vigintiles are significantly different from one another. Thus, the data support the findings from previous research suggesting that the individual’s placement in the relative income hierarchy is strongly negatively correlated with the probability of transiting into sickness absence.

Consequently, an individual’s degree of relative deprivation appears to operate as a major determinant of the hazard of becoming sickness absent. This is consistent with the expectation that an individual’s degree of relative deprivation is a valid indicator of their degree of work-related stress, with negative repercussions on the individual’s (primarily psychosocial) health.

Model 2 is extended with a set of dummy variables capturing the duration of the current employment spell. Comparing the values of the maximized log-likelihood functions reveals that the addition of this information substantially improves the model’s explanatory power. Moreover, the respective values of *ρ* indicate that the importance of unobserved individual characteristics is markedly greater in the conventional model specification (Model 1) than in the duration model (Model 2). This confirms that the duration of uninterrupted employment indeed is an important determinant of an individual’s probability of transiting into sickness absence.

**Figure 3:**
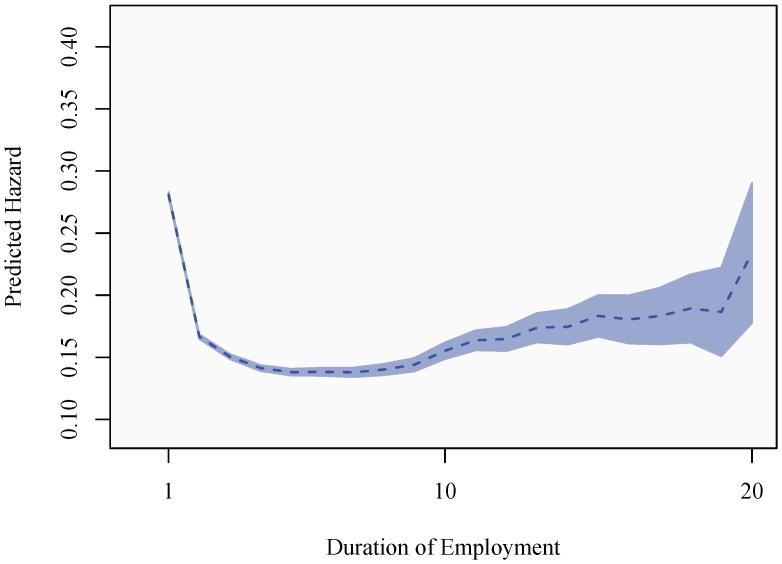
The effect of duration on the sickness absence hazard.

The estimates for the duration dummies (available upon request) indicate a rapidly decreasing probability of sickness absence as the duration of the employment spell increases, until approximately seven years of uninterrupted employment. Past this point, the sickness absence risk gradually increases, yet never reaching the baseline risk experienced during the first year (reference category) of the employment spell. Figure 3 shows the estimated hazard rate (and point-wise 95% confidence intervals) as a function of duration when all explanatory variables are kept constant at their overall means, and only the effect of duration changes. Unlike the raw probability of becoming absent due to sickness depicted in Figure 2, the estimated hazard does not decrease monotonically with duration, but exhibits a roughly u-shaped form. This indicates that the negative effect of duration on the raw hazard can be partly explained by the explanatory variables included in the regression model. In particular, the relative income, which increases with duration and has a negative impact on the hazard, captures a substantial part of the duration dependence. The remaining variations in the hazard function shown in Figure 3 can be interpreted as the effects of unobserved temporal heterogeneity, *i.e*., effects that vary with duration and are not captured by the covariates included in the regression.

As a consequence of including duration dummies, the influence of relative income is considerably moderated, especially for the most relatively deprived. The odds ratios now range from 3.09 (*i.e*., *β* ≈ 1.13) for the lowest category to 0.57 (*i.e*., *β* ≈−0.57) for the highest category, which can be compared with a corresponding range of 3.66 to 0.54 in Model 1. Again, the odds ratios are calculated compared to an otherwise similar individual with a relative income of one. Thus, while the link between relative income and the probability of experiencing sickness absence appears to be robust, the strength of this relation appears to be upwardly biased when failing to account for duration.

**Figure 4:**
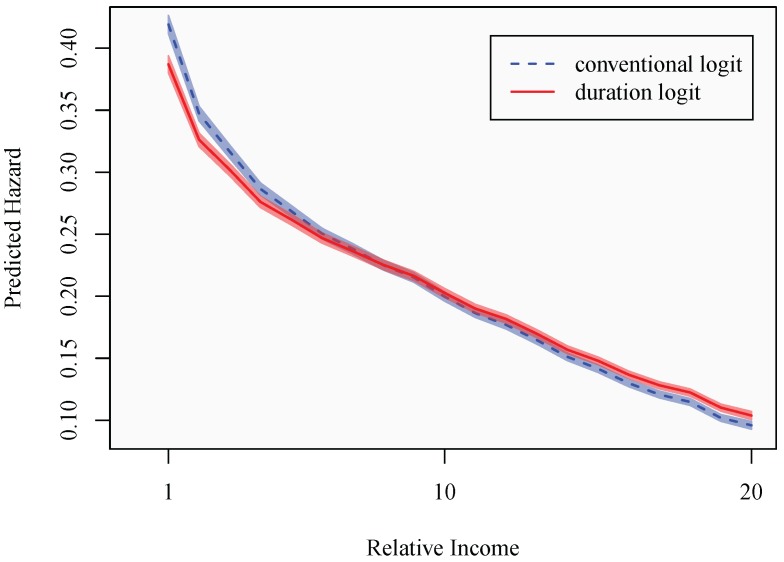
The effect of relative income on the sickness absence hazard.

Figure 4 confirms this, showing predicted hazards as mean of covariates, as well as point-wise confidence intervals, only manipulating the typical individual’s relative income. The hazards are calculated based on Models 1 and 2, confirming that a failure to account for employment duration produces an exaggerated effect of relative income on the sickness absence hazard. In absolute terms, the bias is particularly accentuated at low relative incomes. This suggests that the conventional (binary choice model) approach over-estimates the influence of relative income on the sickness absence propensity among low performers in the labor market. At the other end of the relative income spectrum, the bias serves to underestimate the influence of relative income in models not controlling for duration. When the effect of duration is accounted for, the sickness absence hazard ranges from 0.39 for the typical individual with the lowest relative income (category 1) to 0.10 for the typical individual with the highest relative income (category 20). This shows that relative deprivation has a substantial effect on the sickness absence propensity, even when the effect of duration is taken into account.

Model 3 further controls for aspects relating to the dynamics of the individual’s labor market career. Again, the increase in log-likelihood suggests an improved model fit, also indicated by the decreased value of *ρ*. Compared to Model 2, the relative income effect is hardly affected by these additional control variables, however. The odds ratios range from 3.13 (*i.e*., *β* ≈ 1.14) for the lowest category to 0.58 (*i.e*., *β* ≈−0.55) for the highest category. Again, this can be compared with a corresponding range of 3.66 to 0.54 in Model 1.

While the relevance of controlling for the dynamics of the individual’s labor market career emerges quite clearly from the results of Models 1–3, it remains difficult to discuss the likely mechanisms driving the observed results. The theoretical section postulated a rather complex relationship between various aspects of individual SES and health. Holding other measures of SES constant, it was argued that individuals with more education should enjoy superior health and, consequently, a lower risk of sickness absence.

In order to further investigate the mechanisms determining sickness absence, both income measures are allowed to differ according to the individual’s educational level. More specifically, an individual’s position in the relative income distribution is intended to be informative regarding the degree to which an individual is exposed to psychosocial stress through their labor market performance. To the extent that individuals with low relative incomes are increasingly psychosocially stressed, they should experience poorer health. While individuals in the same relative income position experience similar relative labor market returns, the health advantage should arguably be increasing with the individual’s educational level. More specifically, the more highly educated should enjoy more rewarding jobs, as well as a lower exposure to physically hazardous work environments. However, at the lower end of the relative income spectrum, it is possible that the degree of psychosocial stress is accentuated among the highly educated through the comparatively larger discordance between expected and realized outcome within this group.

**Figure 5:**
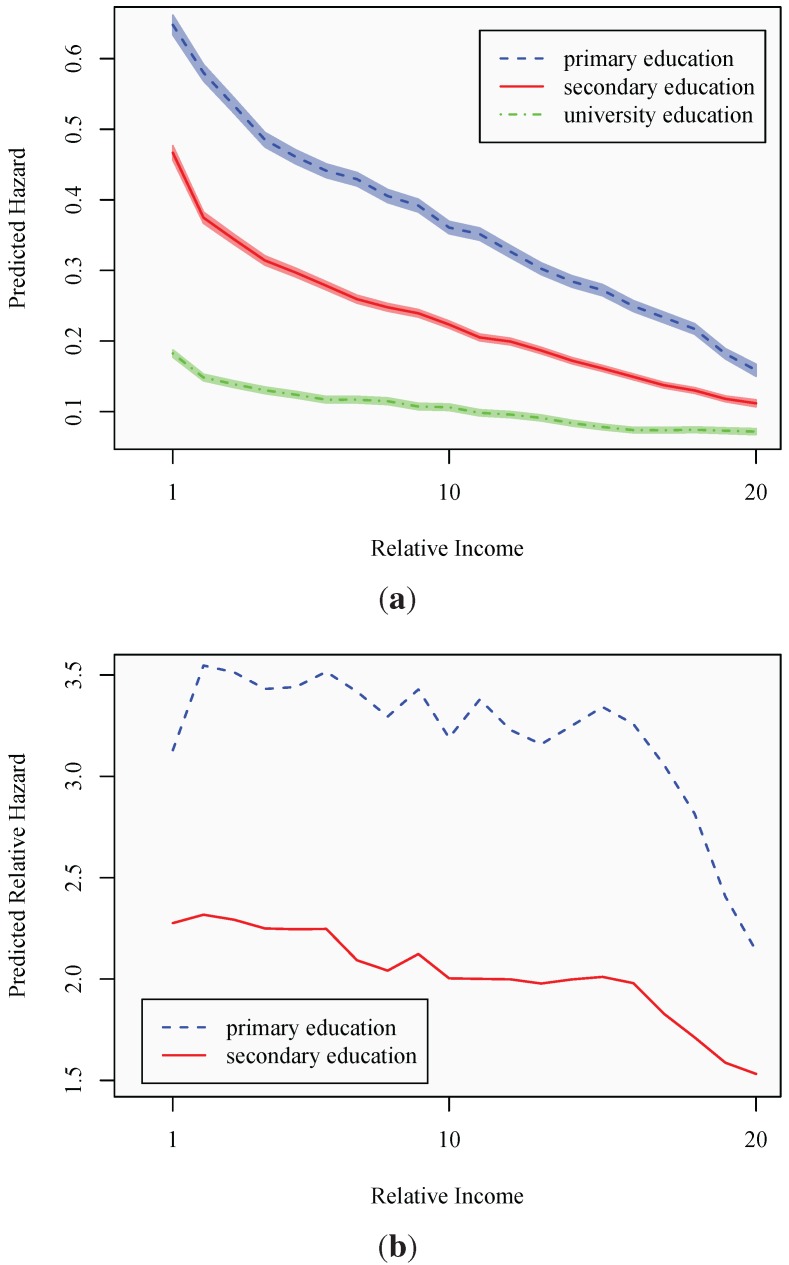
The effect of relative income and education on the sickness absence hazard. (**a**) Relative income and absolute hazard; (**b**) relative income and relative hazard.

Lastly, the *a priori* expectations would suggest that individuals with higher education have superior skills in translating economic resources into health promoting goods. This ability originates through superior knowledge regarding and utilization of health-promoting behaviors, combined with better capabilities to gather and process information. Consequently, individuals may differ in their consumption behavior, with the highly educated being increasingly likely to direct resources, measured through absolute income, towards consumption behaviors that are health promoting. The estimates are presented in Model 4, with baseline effects for both relative and absolute income for individuals with primary education. In Figure 5, predicted probabilities of sickness absence are presented for a typical individual belonging to each respective educational category.

As expected, Figure 5(a) confirms the overall lower hazard of transiting into sickness absence among individuals with higher education. Since the individual’s degree of satisfaction with their labor market returns should be captured by their relative income, this could be indicative of the aforementioned differences in baseline health or suggest differences in working conditions, linked to the individual’s educational level. The figure suggests a consistent decline in the probability of sickness absence with increasing relative income, for all educational levels. In absolute terms, the difference between educational levels, holding relative income constant, is declining with relative income. However, a more relevant way of identifying between-group behavioral differences is arguably as is done in Figure 5(b). Here, the primary and secondary groups’ predicted hazards are expressed relative to the university educated. The figure clearly shows the relative risks of sickness absence among the secondary school educated and, particularly, the primary school educated to be declining substantially at the most elevated relative income levels. More specifically, at relative incomes exceeding 100% (*i.e*., income categories above 13), the likelihood of being absent due to sickness, relative to the university educated, diminishes in an accentuated fashion. This is clearly most pronounced among the primary school educated, whose relative risk of sickness absence declines from around 250% higher to around 140% higher than the hazard of the typical university educated individual in the sample. Furthermore, the declining relative risk is not consistent with the situation displayed at lower relative incomes, where the relative risk appears to hover at a comparatively stable level. As expected, the higher risk of sickness absence experienced by the secondary school educated is consistently less accentuated, but displays a similar pattern.

**Figure 6:**
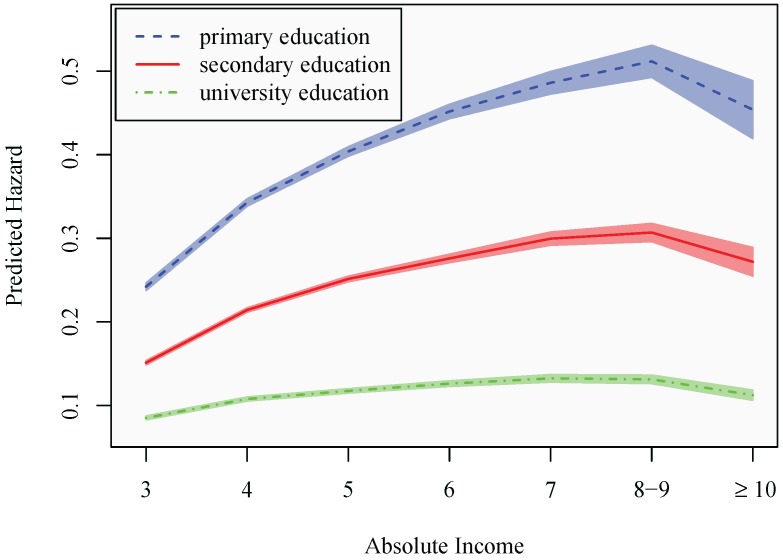
The effect of absolute income on the sickness absence hazard.

Turning to absolute income attainment, the results indicate quite differing influences on the sickness absence hazard, by educational level. Figure 6 shows predicted sickness absence hazards for different levels of absolute income attainment, again at educational level means. The positive association between absolute income attainment and sickness absence propensity that was previously observed is clearly driven by the primary and, albeit to a considerably lesser extent, secondary school educated. Among the university educated, the influence from the individual’s economic resources is seemingly irrelevant. The pattern could suggest fundamentally different consumption behaviors between the social groups, where increasing economic affluence among, in particular, the primary school educated does little to improve their poorer baseline health. Instead, the increasing economic resources appear to be reinforcing the adverse health behavior that is sometimes suggested in the literature.

Out of necessity, certain assumptions regarding the definition of a sickness absence period had to be made when generating the study sample outlined in Section 3. In order to investigate to what extent the results are sensitive to the definition of a sickness absence period, Model 5 considers subsequent years during which the individual is sickness absent as one coherent period of sickness absence. Otherwise, Model 5 is specified in an analogous manner as Model 4, which sees each year with a sickness absence period as a unique event. As confirmed by parameter estimates, as well as predicted hazards, this alternative definition hardly alters the previously obtained results. The same conclusions are reached from Model 6, which is estimated on a sample where only the individual’s first employment spell is included, effectively removing problems caused by repeated spells being interrelated. Again, the model specification is otherwise analogous to Model 4. The rationale for conducting this sensitivity analysis is to test the assumption of conditionally-independent spells. Since many individuals experience multiple employment spells (either ending in sickness absence or being right-censored), the assumption of conditional independence might be violated. Although the inclusion of individual-specific random intercepts into the regression models should suffice to ensure conditional independence among individuals’ (potentially many) spells, estimating a model using one spell per individual only is a viable way of testing the robustness of the results with respect to the independence assumption.

## 5. Conclusions

This study has set out to disentangle the impacts of relative income, absolute income and educational level on the propensity to become absent due to sickness from work. While other studies have examined this topic, to the best knowledge of the authors, none has attempted such a multi-faceted approach.

Our conclusions are at times self-evident and at times somewhat counterintuitive. Among the self-evident is that the amount of time an individual spends working without experiencing sickness absence has an effect on the probability of becoming sick. This may seem obvious, but it has not been controlled for in previous studies. Our results show that without controlling for duration, the effects of other determinants of sickness absence are biased, at least to the extent that they are associated with duration.

The impact factor that we were most interested in was the effect of relative deprivation, measured as relative income. Our results show that relative deprivation is important and that it is important across all educational levels. As relative income increases, the propensity to become absent due to sickness declines, indicating that labor market success is an important factor. The relative relationship between the educational categories is fairly constant, until the absolute highest relative income categories, where a dramatic convergence is observed.

The fact that sickness absence propensity increases as educational level decreases is seen as a sign that individuals from different educational categories have different health-related behavior. We come to this conclusion based on the fact that access to healthcare in Sweden is universal and extremely low-cost, which should eliminate individuals neglecting their health due to economic constraints. As education increases, it can be expected that knowledge pertaining to health promotion also increases, which would produce the education gradient observed. The fact that the absolute highest relative income levels at the primary and secondary educational levels experience dramatic declines relative to university educated individuals may be the result of these individuals belonging to the far right tail of the ability distribution and, therefore, having similar knowledge of health-promoting activities. While we see this as a plausible explanation, we cannot, based on this study, exclude the possibility that selection mechanisms into education could also be to blame. If we assume that health factors, either genetic or evolving in early years, are of importance to educational outcomes, then education may be seen as spurious and the higher propensity for sickness absence at lower educational levels simply the outcome of general health status. Given the size and representativeness of our sample, however, the health pathway to educational attainment would have to be the dominant determinant of educational outcomes for this explanation to totally remove the importance of health-related behavior during adulthood.

In terms of absolute income, we find results that may seem counterintuitive. When controlling for duration, relative income and education, increases in absolute income levels yield increases in the propensity to become absent due to sickness, at least for the primary and secondary educational categories. This is interpreted in much the same manner as the differences between educational groups. As members of the lower educational categories receive greater resources, they may invest these in a greater consumption of health-reducing behavior. As the incomes reach the top in each group, we see that sickness absence propensity decreases, which we interpret as a positive selection, based either on health or intelligence.

Based on the results presented in this study, it appears that all four factors of interest are important, and neglecting to account for any in an analysis of sickness absence can lead to a misinterpretation of the determinants of unscheduled absence from work. Further, it does not appear that a redistribution of economic resources, without a leveling of educational attainment, would have significant impacts on sickness absence behavior.
